# Seizure-Induced Oxidative Stress in Status Epilepticus: Is Antioxidant Beneficial?

**DOI:** 10.3390/antiox9111029

**Published:** 2020-10-22

**Authors:** Tsu-Kung Lin, Shang-Der Chen, Kai-Jung Lin, Yao-Chung Chuang

**Affiliations:** 1 Department of Neurology, Kaohsiung Chang Gung Memorial Hospital, Kaohsiung 83301, Taiwan; tklin@adm.cgmh.org.tw (T.-K.L.); chensd@adm.cgmh.org.tw (S.-D.C.); 2 Center for Mitochondrial Research and Medicine, Kaohsiung Chang Gung Memorial Hospital, Kaohsiung 833, Taiwan; b101101092@tmu.edu.tw; 3 College of Medicine, Chang Gung University, Taoyuan 33302, Taiwan; 4 Institute for Translation Research in Biomedicine, Kaohsiung Chang Gung Memorial Hospital, Kaohsiung 83301, Taiwan; 5 Department of Neurology, School of Medicine, College of Medicine, Kaohsiung Medical University, Kaohsiung 80708, Taiwan; 6 Department of Biological Science, National Sun Yat-sen University, Kaohsiung 80424, Taiwan

**Keywords:** epilepsy, status epilepticus, oxidative stress, antioxidant

## Abstract

Epilepsy is a common neurological disorder which affects patients physically and mentally and causes a real burden for the patient, family and society both medically and economically. Currently, more than one-third of epilepsy patients are still under unsatisfied control, even with new anticonvulsants. Other measures may be added to those with drug-resistant epilepsy. Excessive neuronal synchronization is the hallmark of epileptic activity and prolonged epileptic discharges such as in status epilepticus can lead to various cellular events and result in neuronal damage or death. Unbalanced oxidative status is one of the early cellular events and a critical factor to determine the fate of neurons in epilepsy. To counteract excessive oxidative damage through exogenous antioxidant supplements or induction of endogenous antioxidative capability may be a reasonable approach for current anticonvulsant therapy. In this article, we will introduce the critical roles of oxidative stress and further discuss the potential use of antioxidants in this devastating disease.

## 1. Introduction

Epilepsy is a common neurological disorder and estimated with a prevalence of 0.5–1.5% in the general population varying between developed countries and developing countries [1,2,3]. The term "epilepsy" originated from the Greek verb “epilambanein” (επιλαμϖανειν), which means “to be attacked or to be seized”. Epilepsy as a medical condition is known throughout human history and was described among famous people such as Fyodor Dostoyevsky (Russian writer), Gaius Julius Caesar (Roman general and the great strategist), and Napoleon Bonaparte (French general and Emperor) [4,5,6]. According to the International League Against Epilepsy (ILAE), an epileptic seizure is caused by abnormal, excessive, and synchronous neuronal activity in the different brain areas and presenting various signs and/or symptoms accordingly [7]. Epilepsy is typified by repeated seizures and the aberrant firing of neurons located mainly in the cerebral cortex and often under unprovoked conditions. The burst firing neurons accompanied with epileptic discharges could result in a variety of changes at the cellular level, e.g., activation of glutamate receptors, alteration of γ-aminobutyric acid (GABA) receptor, activation of cytokine expression, increased oxidative stress, modification of neurogenesis, adjustment of plasticity or stimulation of some late cell death pathways [8,9,10].

Status epilepticus, defined with prolonged or continuous epileptic seizures, is a neurological emergency associated with significant morbidity and mortality [11,12,13]. Clinical studies revealed that patients with epilepsy are often associated with behavioral and cognitive decline from multifactorial contributions [14,15,16]. About 4–16% of people with epilepsy have experienced at least one attack of status epilepticus [17]. A recent study showed status epilepticus with prominent motor phenomena was 24 per 100,000 adults per year [18]. Status epilepticus has a 10–20% mortality in previous studies [13,17], although more rapid treatment regimens at an early stage reduce disease-related mortality in recent studies [12,19]. Human and animal studies also revealed that status epilepticus causes substantial cerebral damage, raises the risk to develop succeeding epileptic episodes, and accompanies with a distinctive pattern of neuronal death in the hippocampus, such as in dentate gyrus hilus, CA3, and CA1 subfield [20,21].

It was gradually recognized that excessive oxidative stress plays a crucial role in the pathophysiology of status epilepticus [22]. While in prolonged seizure or status epilepticus, reactive oxygen species (ROS) are generated mainly by nicotinamide adenine dinucleotide phosphate (NADPH) oxidase through NMDA receptor activation [23,24]. The inflammatory response following status epilepticus causes activation of inducible nitric oxide synthase (iNOS) and iNOS-derived nitric oxide (NO) reacts with O_2_^-^ to form the peroxynitrite and contributes the severity of oxidative stress in kainic-acid induced experimental status epilepticus in our previous studies [25,26,27,28]. Several studies with a various animal model of status epilepticus such as pilocarpine-induced, pentylenetetrazole-induced, diisopropylfluorophosphate, lithium-pilocarpine model, also demonstrated the critical role of NO-related pathway and neuro-inflammation contributing to neuronal damage [29,30,31,32,33,34,35,36].

Excessive production of ROS/reactive nitrogen species (RNS) can damage macromolecules in cells such as with lipid peroxidation, DNA damage, oxidative damage with enzyme inhibition and result in neuronal death [22]. As excessive ROS/RNS may affect neuronal fate through diverse mechanisms, modification of those detrimental pathways may have beneficial clinical implications. In this review, we will focus on the critical role of ROS/RNS in status epilepticus which can further impair mitochondrial function, enhance the severity of oxidative stress, and cause neuronal damage or death. The potential use of antioxidants in this devastating disease will be further discussed.

## 2. The Critical Cole of ROS/RNS in Status Epilepticus

It is known that the burst-firing neurons with epileptic discharges in prolonged seizure can lead to substantial changes in neuronal cells. These include toxic activation of glutamate receptors, decreasing the reuptake of glutamate, alteration of GABA receptor, an upsurge of cytokine expression, escalation of oxidative stress, modification in neuroplasticity, and later to initiate cell death pathways [37].

Diverse sources of ROS exist in living cells that include 5-lipoxygenase, NADPH oxidase, cytochrome P450 enzymes, and mitochondria [38,39,40]. Balanced ROS are indispensable in the preservation of redox homeostasis and making numerous cellular signaling pathways functional [41]. Living organisms can generate unwarranted ROS under various stressful conditions such as with cytokine stimulation, serum deprivation, and hypoxia [40,41]. To keep a low level of ROS/RNS is vital for continuing the process of regular neuronal function [42]. Overproduction of ROS/RNS may proceed with a detrimental effect and generate wide-ranging damage to lipid membranes, protein components, and DNA structures that can evolve to cell death. This is implicated in the pathogenesis of various neurodegenerative disorders such as Alzheimer’s disease (AD), Parkinson’s disease (PD), and Huntington’s disease (HD) [43,44,45]. This is no exception for seizure disorder, one of the common neurological disorders, which also need to deal with excessive oxidative stress.

Conventionally, it was assumed that mitochondria are the main source of ROS during epileptiform activity [46,47]. It was gradually appreciated with recent studies that in prolonged seizure or status epilepticus, ROS are generated mainly by NADPH oxidase through NMDA receptor activation [23,24]. Using live-cell imaging techniques in neuron-glia cultures, it was demonstrated that prolonged seizure-like activity raises ROS generation in an NMDA receptor-dependent manner rather than calcium- and mitochondria-dependent ROS pathway [23]. It was reported that ROS derived from NADPH oxidase in the pilocarpine-induced temporal lobe epilepsy and apocynin, an NADPH oxidase inhibitor, attenuated ROS production and neurodegeneration from status epilepticus [48] and prevent seizure-induced neuronal death [49]. Using the perforant path model of epilepsy, inhibition ROS production by 4-(2-Aminomethyl)benzenesulfonyl fluoride hydrochloride (AEBSF), another NADPH oxidase inhibitor, significantly lessened seizure-induced cell death [50]. In a recent study, it was demonstrated the critical role of NMDA receptor-mediated NADPH oxidase-induced oxidative stress and targeting NADPH oxidase may offer an innovative treatment for epilepsies [51]. In a human study with surgically resected epileptic tissue from drug-resistant patients, the hippocampus showed cytoplasmic positivity for p47(phox) and p67(phox) in neurons and glial cells which indicates the activation of NOX2 [52]. Inhibition of NADPH oxidase activation by apocynin involves hippocampal neurogenesis which revealed increased neuronal survival and neuroblast production [53]. It was reported that pilocarpine rats had increased NADPH oxidase 2 expressions and decreased superoxide dismutase (SOD) expression [54]. With in vivo and in vitro pilocarpine model of epilepsy, hyperactivation of NMDA receptors and subsequent activation of NADPH oxidase are crucial contributors for the development of epileptogenesis [55]. The oxidative stress induced by NADPH oxidase activation also plays a pivotal role in the pentylenetetrazol-induced kindling process as well as in pentylenetetrazol kindling-induced hippocampal CA1 autophagy [56]. A study with a combination of antioxidant therapy including NADPH oxidase inhibition and the endogenous antioxidant system activation such as nuclear factor erythroid 2-related factor 2 (Nrf2) can prevent epileptogenesis and modifies chronic epilepsy [57]. These studies revealed the critical role of NADPH oxidase, particularly, NADPH oxidase 2, in oxidative stress-induced neuronal damage and involved neurogenesis, autophagy and epileptogenesis as well.

The production of RNS has closely related ROS. RNS is derived from nitric oxide (NO) and with superoxide (O_2_), a reactive oxygen species, to form the peroxynitrite. Peroxynitrite, a highly reactive species, can directly or indirectly react with other cellular components such as DNA, lipids or proteins and induced neuronal death [58]. We have shown before the findings of upregulation of iNOS in the hippocampal CA3 subfield following kainic acid-induced experimental status epilepticus in the rat [27]. Experimental findings revealed the increased level of O_2_^−^ and peroxynitrite and sequential changes in time were well correlated accordingly which lead to mitochondrial apoptosis in the hippocampal CA3 subfield [26]. We further provide evidence that activation of NF-kappaB (NF-κB), a transcriptional factor critical for inflammatory genes expression, heightens iNOS gene expression in hippocampal CA3 neurons following status epilepticus in an animal model [25].

It was generally appreciated that glutamate-mediated excitotoxicity causing necrotic changes in the cerebral cortex are the major morphological findings among dying neuronal cells after status epilepticus [59]. There has been evidence that revealed apoptotic cell death also plays a crucial role in seizure-induced brain damage in animal models of experimental status epilepticus [27,60,61,62]. It has long been aware that oxidative stress, apoptosis, and neuroinflammation have a key role in various neurological diseases such as ischemic stroke, neurodegenerative diseases or epilepsy [63,64,65,66,67]. Apparently, neuroinflammation and oxidative stress are mutually affected with each other, contribute to immediate and longstanding sequelae of status epilepticus, and indicate a potential therapeutic target.

Several inflammatory mediators were shown to have critical roles in status epilepticus such as Toll-like receptor 4 (TLR4), high mobility group box 1(HMGB1), NF-kB, NLRP3, and iNOS which are induced during status epilepticus and predict the development of neuronal cell loss, cognitive deficits, mortality, the progress of chronic epilepsy [68]. Mounting evidence revealed the signaling pathways involving neuroinflammation affecting status epilepticus. TLR4 and receptor for advanced glycation end product (RAGE) are activated by HMGB1 and critical for seizure generation [69,70]. IL-1 receptor type 1 (IL-1R1)/TLR4 pathway is a potential therapeutic target for disease-modifications in patients with epilepsy [71]. Inhibitors of the IL-1R/TLR signaling exert the anticonvulsant effects in various seizures models which indicate the potential to reduce seizure frequency in currently pharmaco-resistant epilepsies [72]. Seizure-induced neuroinflammation impairs the integrity of the blood-brain barrier (BBB) [73]. Inhibition of the prostaglandin E2 receptor 2 can reduce inflammation, restore BBB function, and decrease mortality following status epilepticus [74]. It was realized that excessive oxidative stress and neuroinflammatory response occur together during status epilepticus and persist afterward. These results may further damage the mitochondrial function and aggravate the severity of oxidative stress. We will further discuss mitochondrial dysfunction after status epilepticus under these signaling cascades toward the neuronal death in the next section.

## 3. Mitochondrial Dysfunction Further Aggravates the Extent of Oxidative Stress Following Status Epilepticus

Mitochondria are membrane-bound cellular organelles widely distributed in the cytoplasm of cells in most eukaryotic organisms. Emerging studies revealed the multifaceted roles of mitochondria which involve various pathophysiological functions such as energy production, ROS generation, apoptotic process, mitochondrial biogenesis, dynamics, mitophagy, and inflammation [75]. Mitochondrial oxidative phosphorylation comprises five enzyme complexes placed in the mitochondrial inner membrane [76,77,78]. These are NADH-ubiquinone oxidoreductase (Complex I), succinate-ubiquinone oxidoreductase (Complex II), ubiquinone-cytochrome c oxidoreductase (Complex III), and cytochrome c oxidase (Complex IV) which work together to produce a proton motive force and drive the generation of ATP by complex IV (F1F0-ATP synthase).

As status epilepticus can cause excessive oxidative stress accompanying the neuroinflammatory response and augment the formation of peroxynitrite from NOS II derived NO and O_2_^−^ which may impair the mitochondrial function. Previous studies revealed that prolonged epileptic seizures will alter the redox status, reduce the production of ATP, and lead to energy failure in the brain [79,80]. There has been evidence to show mitochondrial dysfunction in epilepsy in animal and human studies [28,81,82,83,84]. We have shown before that in kainic acid-induced status epilepticus, enzyme assay for the mitochondrial respiratory chain showed a notable depression of the activity of Complex I + III in the dentate gyrus and subfield of CA1 and CA3 in the hippocampus [28]. In contrast, the activities of Complex II + III and Complex IV remained unchanged. These findings were accompanied by swelling of mitochondrial spaces including cristae and a variable degree of mitochondrial membrane disruption. These findings stressed the pivotal role of Complex I dysfunction in mitochondria and damage of ultrastructure in the mitochondria membrane in the hippocampus of kainic acid-induced status epilepticus in the rat [28]. In a pilocarpine-induced status epilepticus, selective decline of Complex I and IV function in the respiratory chain was observed in hippocampal CA1 and CA3 subfields and suggested that seizure activity can downregulate the mitochondrial-encoded enzyme’s activity of oxidative phosphorylation [85]. Inhibition of mitochondrial Complex I activity was also reported in other studies using different animal models [81,83]. In a human study, it was observed that mitochondrial Complex I dysfunction in the CA3 subfield in patients with refractory temporal lobe epilepsy [84]. Although the underlying mechanism is not well elucidated, it appeared that Complex I of the mitochondria is more vulnerable to ROS and RNS than in other respiratory chain complexes [86].

It is known that status epilepticus can cause mitochondrial respiratory chain dysfunction and result in energy failure. These consequences can further aggravate the severity of oxidative stress and cause neuronal damage or death in the hippocampus. Although glutamate-mediated excitotoxicity often causes necrotic changes in the cerebral cortex after status epilepticus [59], several studies including ours showed that apoptotic cell death also plays an important role in seizure-induced brain damage in experimental status epilepticus [27,60,61,62]. The existence of apoptosis may lend some time to intervene and lessen neuronal damage, such as with antioxidant treatment. The apoptosis process includes the successive activation of a stream of cysteine proteases (caspases) [87]. A long list of apoptosis-related proteins from mitochondria was entailed such as apoptosis-inducing factor (AIF), cytochrome c, endonuclease G, Smac/DIABLO, and HtrA2/OMI [37,88]. Cells also comprise both pro-apoptotic and anti-apoptotic “Bcl-2 family” proteins and are engaged to the apoptotic pathway under various clinical conditions including brain disease. Detailed apoptosis mechanisms are beyond the scope of this review; some of the excellent reviews available can be helpful for readers to gain a further understanding [88,89,90,91,92].

Except for the well-known pathways involving mitochondria-related oxidative stress, apoptosis, and neuronal damage, the emerging role of the sirtuin family, or the special diet formulation such as ketogenic diet (KD), are also revealed as crucial players in status epilepticus and worth mentioning here. In mammals, the sirtuin family include seven sirtuins: sirtuin1–7, and belong to a class of nicotinamide adenine dinucleotide (NAD)-consuming enzymes that are involved in various biological pathways [93]. The beneficial effect of sirtuins on longevity is related to the capability to modulate on the metabolic pathways which present a potential target to treat human diseases [93,94]. Limited studies concerning the sirtuin family and status epilepticus were reported. It was shown that sirtuin1 activation increases the peroxisome proliferator-activated receptor gamma coactivator 1-alpha (PGC-1α) expression and enhances mitochondrial antioxidant system in status epilepticus [95]. We have demonstrated that the downregulation of sirtuin1 can reduce PGC-1α expression, impair mitochondrial biogenesis, augment Complex I dysfunction, heighten the extent of oxidized proteins, increase caspase-3 expression, and promote neuronal cell damage in the hippocampus in kainic acid-induced status epilepticus [96]. It was also revealed that microRNA-199a-5p regulates the sirtuin1-p53 pathway in pilocarpine-induced status epilepticus and targeting of microRNA-199a-5p exerts a beneficial effect in seizure disorder [97]. In a recent study, it was shown that sirtuin3, a major mitochondria NAD^+^-dependent deacetylase, through regulating manganese SOD to lessen excessive oxidative stress from mitochondria can reduce neuronal damage in hippocampal cells after status epilepticus [98]. With these studies, it is noteworthy to further explore the vital role of the sirtuin family in seizure disorders, and this may open an avenue to develop innovative therapy in epilepsy in the future.

The KD is a special diet formulation with high fat, low carbohydrate, controlled protein regime that has been used since one hundred years ago for the treatment of epilepsy [99]. The mechanisms of KD may act through the effects of glucose restriction with ketones formation and interactions with receptors, channels, and metabolic enzymes [100,101]. It can reduce mitochondrial ROS/RNS due to the alteration of the source of energy by using more fat-derived ketone bodies and with less consumption from carbohydrates [102,103,104]. There are several different forms of the ketogenic diet such as the classical diet, medium-chain triglyceride diet, modified Atkins diet, and modified ketogenic diet, and low glycaemic index treatment [100,101,102,103,104,105]. These diet formulas may help to reduce the number or severity of seizures in patients with poorly controlled seizures after two more anti-epileptic drugs used. KD is a well-known therapeutic option for children with poor control epilepsy [105]. Mounting evidence also reveals the effectiveness of KD for adults with status epilepticus which denotes the usefulness through dietary adjustment [106,107,108]. The diet modification offers another treatment option in patients with refractory epilepsy and warrants to further testify the crucial role of critical condition as in status epilepticus.

**Figure 1:**
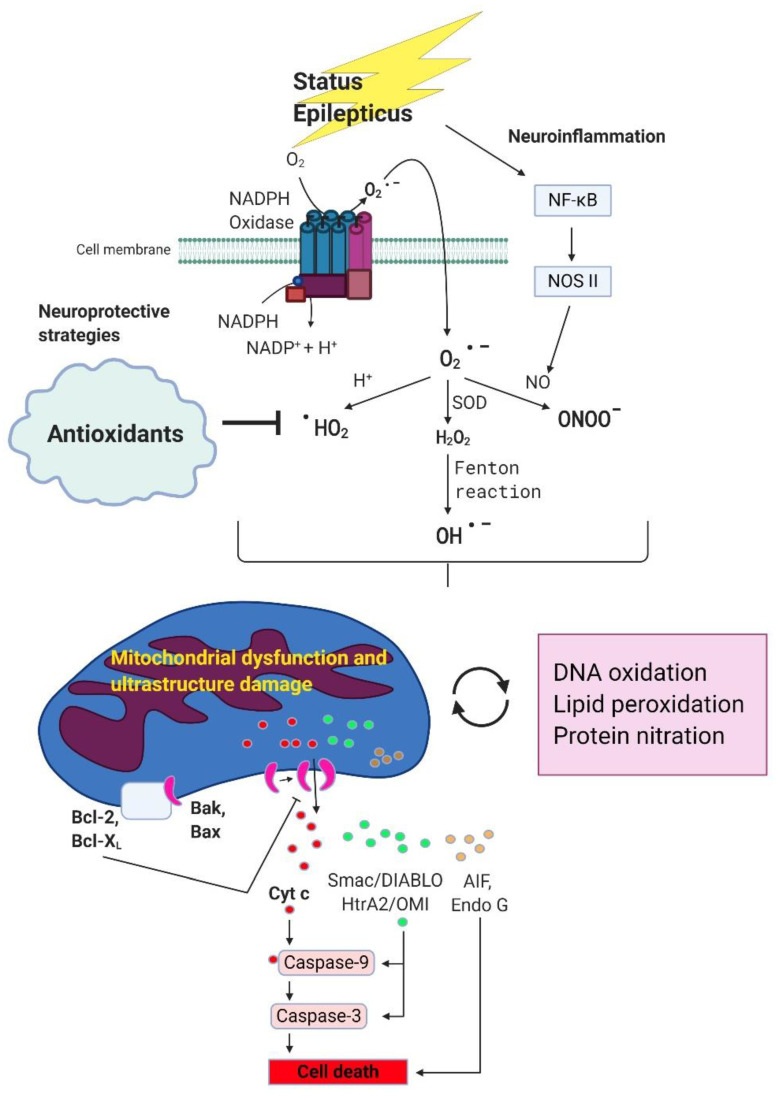
The potential beneficial effects of antioxidants on status epilepticus induced oxidative stress, mitochondrial damage and neurodegeneration. During status epilepticus, superoxide are generated mainly by nicotinamide adenine dinucleotide phosphate (NADPH) oxidase through NMDA receptor activation. Once produced, superoxide can interact with various molecules and further generate secondary radicals. Superoxide can react with nitric oxide and produce peroxynitrite. Superoxide can also be converted into hydrogen peroxide by superoxide dismutase, and then to hydroxyl radicals under the famous Fenton reaction. Interaction of superoxide with protons can produce hydroperoxyl radicals. All these reactive oxygen species/reactive nitrogen species (ROS/RNS) are highly reactive and can cause oxidative stress via alterations of macromolecules. Overproduction of these ROS/RNS due to prolonged epileptic seizures will lead to alteration of mitochondrial redox status, Complex I dysfunction and ultrastructure damage in mitochondria membrane, reduce ATP production and eventually the induction of mitochondrial dependent apoptotic pathway involving the release of a serious of apoptosis-related proteins from mitochondria intermembrane space including apoptosis-inducing factor (AIF), cytochrome c, endonuclease G, Smac/DIABLO and HtrA2/OMI. Protection of mitochondria from bioenergetic failure and oxidative and nitrosative stress with antioxidants may be considered as a potential neuroprotective strategy in neuronal degeneration associated with status epilepticus.

It is crucial to deal with excessive ROS production from NADPH oxidase caused by NMDA receptor activation in prolonged seizure or status epilepticus [23,24]. The oxidative stress may further impair mitochondrial ultrastructure of the membrane and mitochondrial respiratory chain and aggravate the severity of oxidant damage to neuronal cells [37,109]. In Figure 1, we briefly sketch the signaling pathway about status epilepticus causing the excessive generation of ROS and further aggravating mitochondrial function, increasing the severity of oxidative stress, and causing neuronal apoptotic death. Except for the anticonvulsants for status epilepticus, adding antioxidants in this scenario may have the potential to improve the therapeutic effect. We will further discuss the significance of antioxidants used in status epilepticus in the following section.

## 4. The Potentials of Antioxidants in Status Epilepticus 

As status epilepticus or prolonged seizure can cause excessive oxidative stress with the various sequential molecular events to cause neuronal damage. Apart from using antiepileptic drugs to reduce or stop the seizure attack, adding antioxidants may have some potential effects to lessen neuronal injury from excessive oxidative stress. Although there are many new antiepileptic drugs with varying mechanisms developed and available in recent decades, more than one-third of patients remained poorly controlled in seizure frequency [110]. It is obvious that there are still unmet needs for drug-resistant epilepsy which highlights the demand for an innovative and maybe an unconventional approach for those patients [111,112]. Studies with drugs or chemical compounds possessing antioxidant characters used in animal models of status epilepticus were selected to discuss and may provide some clues in this regard. However, we do not intend to make a comprehensive review for all kind of antioxidants in epilepsy studies but just to offer a perspective concerning the potential and possibility among drugs or chemical compounds which may be used concurrently with the antiepileptic drugs to further decrease seizure occurrence and improve the outcome of epilepsy. To consider the significance of translation medicine in preclinical studies, we also searched from ClinicalTrials.gov for some of these antioxidants to offer information on the potential clinical use in brain diseases including epilepsy. Selected drugs or chemical compounds with antioxidant characters used in a various animal model of status epilepticus will be presented in Table 1. Relevant information for clinical trials in these compounds and drugs is included and the identifier numbers of ClinicalTrials.gov for epilepsy are provided for readers to more easily locate.

N-acetylcysteine (NAC) or acetylcysteine, a medication often used to loosen thick mucus in individuals with chronic lung disease, can replenish glutathione stores, help the formation of glutathione in the body and exert the antioxidative effect [137,138]. NAC was found to be safe with favorable evidence in many psychiatric and neurological diseases in a systemic review [139]. In pentylenetetrazole-induced seizures, adding NAC exhibited an anticonvulsant effect [113]. More than 30 clinical trials concerning “brain disease and N-acetylcysteine” were searched from ClinicalTrials.gov. Brain diseases include various neurodegenerative diseases, traumatic brain injury (TBI), epilepsy, brain tumor, or vascular diseases. However, no data available from epilepsy and N-acetylcysteine trial due to no eligible subjects located.

Ubiquinone, also known as coenzyme Q, is a coenzyme family and widely distributed in living organisms. In the human being, the most common form is coenzyme Q10_._ As one component in the electron transport chain, ubiquinone plays a crucial role in aerobic cellular respiration and produces ATP for energy need in the body. It also is an essential antioxidant and a modulator of the permeability transition pore and critical in various pathophysiological effects [140,141,142]. In pilocarpine-induced seizures, coenzyme Q10 lessened the severity of oxidative stress. Furthermore, it enhanced the antiepileptic effects of phenytoin treatment [114]. With coenzyme Q10 combined with minocycline in pentylenetetrazol-induced epilepsy, the ability to reverse oxidative damage and restore mitochondrial enzyme complex activities is better than control and individual effect of coenzyme Q10 or minocycline alone [115]. There were 35 clinical trials that were found concerning “brain disease and coenzyme Q10” that were found on ClinicalTrials.gov. Various brain diseases patients recruited for the study include migraine, stroke, AD, PD, HD, ataxia, mitochondrial disease, and epilepsy. One study including coenzyme Q10 as one of multi-vitamin supplementation in epilepsy subjects is still recruiting and no result available yet.

Naringenin is a flavanone and was found in various fruits and herbs [143]. It has significant antioxidant properties, affected plasma lipid levels and antioxidant activity [144], protected mice against radiation-induced DNA damage [145], or defended ferric iron-induced oxidative damage in HepG2 cells [146]. In pilocarpine-induced status epilepticus, reduction of antioxidant enzymes such as catalase, superoxide dismutase (SOD), and glutathione reductase was found in the hippocampus, and naringenin can reduce ROS formation, restore antioxidant enzymes and lessen seizure severity [116]. No clinical trial was found regarding “brain disease and Naringenin” from ClinicalTrials.gov.

EUK-134 is a salen-manganese complex with potent catalase/SOD activity and exerted beneficial effect to counteract excessive oxidative stress in ischemic brain injury [147]. It was able to extend the life-span in Caenorhabditis elegans by augmenting the natural antioxidant defenses system [148]. In kainic acid-induced seizures, EUK-134 showed a significant reduction of protein nitration, NF-κB-binding activity, and neuronal damage from seizure activity over the limbic system [117]. This may imply the potential of EUK-134 to prevent excitotoxic neuronal injury from various neurological diseases including epilepsy. No clinical trial was found about “brain disease and EUK-134” from ClinicalTrials.gov.

Pioglitazone, a peroxisome proliferator-activated receptor-gamma (PPARγ) agonist used clinically as an anti-diabetic medication, is known to exert various biological functions such as reducing inflammation and oxidative injury and may use for stroke or other neurodegenerative diseases [149]. We have shown before that pioglitazone can exert anti-oxidative and anti-apoptotic effects against ischemic neuronal injury [150]. We also demonstrated that pioglitazone decreased reactive oxygen species, increased mitochondrial uncoupling protein 2 (UCP2) expression, lessened mitochondrial apoptotic process, stabilized the mitochondrial transmembrane potential, and decreased neuronal death in the hippocampus following kainic acid-induced seizures [119]. Pioglitazone also exerts a beneficial effect over genetically epileptic EL mice with a reduction of inflammatory responses in the brain [118]. There are 15 clinical trials concerning “brain disease and pioglitazone” that were searched from ClinicalTrials.gov. The brain diseases include various neurodegenerative diseases, ischemic and hemorrhagic stroke, brain tumor, atherosclerosis, adrenomyeloneuropathy, Friedreich’s Ataxia, and Ataxia-Telangiectasia (AT). No clinical trial involving epilepsy and pioglitazone was found in ClinicalTrials.gov.

Semicarbazide is a water-soluble white solid derivative from urea with the formula OC(NH2)(N2H3). Aryl semicarbazones, the product of semicarbazide, was found to have excellent activity as an anticonvulsant with low neurotoxicity in experimental animals [151]. It can prevent seizure-induced lipid peroxidation and augment antioxidant enzyme activities such as SOD and glutathione peroxidase (GSH-Px) in the brain homogenate of mice [120]. No clinical trials concerning “brain disease and aryl semicarbazones” was found in ClinicalTrials.gov.

α-lipoic acid is a naturally occurring compound and is critical for energy metabolism, redox regulation, and cell signaling [152]. In a systemic review concerning the use of α-lipoic acid for the treatment of psychiatric and neurological conditions, it revealed that α-lipoic acid improves schizophrenia symptoms with fewer side effects from antipsychotic medication. It also showed the effectiveness in the prevention of Alzheimer’s disease progression and the ability to improve clinical parameters and oxidative imbalance in stroke patients [153]. In a clinical trial, α-lipoic acid was found to stimulate cAMP production in healthy control and patients of multiple sclerosis [154]. α-lipoic acid takes part in mitochondrial oxidative metabolism and acting as biological antioxidants with potential therapeutic use in various chronic diseases [155,156]. It was shown in pilocarpine-induced status epilepticus that α-lipoic acid decreased seizure episodes by reducing oxidative stress in rat hippocampus and striatum [121,122]. There are 11 clinical trials concerning “brain disease and α-lipoic acid” that were found from ClinicalTrials.gov. The brain diseases include AD, PD, mucopolysaccharidosis disorders, ischemic stroke, brain injury, adrenomyeloneuropathy, and progressive supranuclear palsy (PSP). No clinical trial concerning epilepsy and α-lipoic acid was found in ClinicalTrials.gov.

Melatonin is a widely existing molecule in living organisms implicated in various biological and physiological effects among different tissues or organs. It possesses the capability to cross the BBB, exerts notable antioxidant effects, performs as a free radical scavenger, increases antioxidant enzymes, lessens mitochondrial electron leakage, and interferes with pro-inflammatory signaling pathways [157]. These effects of melatonin highlight the potential clinical use in various neurodegenerative diseases such as AD, PD, amyotrophic lateral sclerosis (ALS), and multiple sclerosis (MS) [158,159]. In pentylenetetrazole-induced seizures, treatment with melatonin notably altered neurotransmitters and nitrite levels, decreased seizure occurrence, and mortality. The results indicate that the anticonvulsant property of melatonin involves both brain amino acids and NO production [124]. In another study, it was revealed that co-treatment with melatonin in kainic acid-induced seizures, it can lessen the hydroxyl radicals, reduce the damage of mtDNA, decrease lipid peroxidation in the brain, hence lessen the severity of seizures from kainic acid [123]. More than 50 clinical trials concerning “brain disease and melatonin” were found from ClinicalTrials.gov. Brain diseases include various neurodegenerative diseases, traumatic brain injury, epilepsy, brain tumor, cerebrovascular diseases, premature birth-related brain injury, migraine, sleep disorders. Four trials concerning “epilepsy and melatonin” were found for various epilepsy patients including Lennox-Gastaut Syndrome.

α-tocopherol, a type of vitamin E, is engaged in various biochemical processes closely related to lipid and lipoprotein homeostasis. It functions as a hydroperoxyl radical scavenger and is critical in a variety of oxidative stress-related disease conditions in humans [160]. In pilocarpine-induced status epilepticus in rats, it was revealed that pretreatment with α-tocopherol reduced oxidative stress and partially reduced chaperone-mediated autophagy in the hippocampus at 1 day after status epilepticus versus vehicle [125]. With two different chemical compounds, methylmalonic acid and pentylenetetrazol-induced seizure, both revealed the increased content of thiobarbituric acid-reactive substances (TBARS) and total protein carbonylation in striatum and α-tocopherol showed the ability to attenuate the severity in a dose-dependent manner [126]. There are 3 clinical trials concerning “brain disease and α-tocopherol” were found from ClinicalTrials.gov. The brain diseases include premature birth-related intraventricular hemorrhage, common chronic health conditions including migraine and sleep disorders, and dietary habits and consumption including check blood α-tocopherol level in dementia patients (ClinicalTrials.gov Identifier: NCT01193270, NCT02758990, NCT03794141). No clinical trial concerning epilepsy and α-tocopherol was found in ClinicalTrials.gov.

Ascorbic acid, also known as vitamin C, is found in various foods and used as a dietary supplement in daily life. Ascorbic acid is involved in various physiological functions of the body including the nervous system, such as supporting the structure of the neurons, engaging the processes of differentiation, maturation, and survival in the neuron, and aiding and modulating the synthesis of neurotransmission [161]. Ascorbic acid is crucial to maintain the balance of redox state and possesses the potential effect to treat various neurodegenerative diseases, autoimmune diseases, and cancer [161,162,163]. In pilocarpine-induced status epilepticus, it was revealed that ascorbic acid delays the onset of seizure and reduces the mortality rate, and decreases lipid peroxidation levels [127]. In another study, ascorbic acid can restore the decrease of glutathione levels and counteract the increased lipid peroxidation level caused by penicillin-induced epileptiform activity [128]. There are 17 clinical trials concerning “brain disease and ascorbic acid” that were found from ClinicalTrials.gov. The brain diseases include AD, PD, ischemic and hemorrhagic stroke, brain tumor, carotid atherosclerosis, ischemic encephalopathy, hepatic encephalopathy, cerebral palsy, attention deficit hyperactivity disorder (ADHD), and epilepsy. There is one study concerning epilepsy and ascorbic acid entitled “Trial of Vitamin C as Add on Therapy for Children With Idiopathic Epilepsy”, but no result available until now.

Selenium is a trace element and an essential micronutrient for animals. It is integrated into antioxidant enzymes such as glutathione peroxidases (GSH-Px) and thioredoxin reductases (TrxR) and plays an important role in normal brain function [164,165]. Deficiency of selenium may involve with mentality decline and selenoproteins are beneficial to neurodegenerative diseases such as AD, PD, or HD through redox signaling regulation [164,165]. Selenium deficiency also involves the risk of seizures attack and supplementation of selenium may reduce seizures [166]. Increased nitrosative/oxidative stress in hippocampal tissue such as enhanced iNOS expression along with TrxR activity induction have been revealed in a pentylenetetrazol-induced seizure. Selenium treatment helped to reduce the expression of 4-hydroneonenal, nitrotyrosine, iNOS, and concomitantly normalized TrxR and HO-1 activity. Adding sildenafil, a selective phosphodiesterase 5 inhibitor, can further enhance these effects [129]. It was also reported that selenium with topiramate, an antiepileptic drug, has protective effects on the pentylenetetrazol-induced seizure by inhibiting free radicals and supporting antioxidant redox systems [130]. There are 12 clinical trials concerning “brain disease and selenium” that were found from ClinicalTrials.gov. The brain diseases include phenylketonurias, AD, HD, ischemic and hemorrhagic stroke, carotid atherosclerosis, cerebral palsy, and epilepsy. Two trials concerning “epilepsy and selenium” were found but without final results available till now.

Resveratrol, a nonflavonoid polyphenol, is mainly found in red grapes/wine [167]. It can offer protective effects against various cardiovascular diseases, neurodegenerative diseases, stroke, and epilepsy through diverse mechanisms [167,168,169,170,171]. In a lithium-pilocarpine-induced status epilepticus, it was shown that the application of resveratrol can reduce oxidative stress and mitochondrial dysfunction. This denotes the potential of resveratrol, combined with antiepileptic drugs, to target oxidative stress and may provide a further beneficial effect to control epilepsy [131]. It is also shown in kainic acid-induced status epilepticus that in addition to lessening neurodegeneration and aberrant neurogenesis, resveratrol also suppresses the severity of oxidative stress and inflammation in the hippocampus [132]. There are 16 clinical trials concerning “brain disease and resveratrol” that were found from ClinicalTrials.gov. The brain diseases include mainly neurodegenerative diseases such as AD, PD, HD, Friedreich ataxia, and hypoxic brain. No clinical trial concerning epilepsy and resveratrol was found in ClinicalTrials.gov.

RTA 408 (omaveloxolone) holds the characters of both antioxidative and anti-inflammatory activities [172,173], and possesses the capability to improve mitochondrial bioenergetics [174]. The mechanism of RTA 408 has been suggested to involve a combination of activation of the antioxidative transcription factor nuclear factor erythroid 2-related factor 2 (Nrf2) and inhibition of the pro-inflammatory transcription factor NF-κB [172]. In a kainic acid-induced status epilepticus in rats, RTA 408 inhibits kelch like ECH associated protein 1 (KEAP1) expression and activates Nrf2 to increase endogenous antioxidant and eventually reduce cellular ROS production and neuronal death. RTA 408 also significantly reduced the frequency of late spontaneous seizures following status epilepticus [133]. To date, one clinical trial concerning “brain disease and RTA 408” was found from ClinicalTrials.gov (ClinicalTrials.gov Identifier: NCT02255435). The title is “A Phase 2 Study of the Safety, Efficacy, and Pharmacodynamics of RTA 408 in the Treatment of Friedreich’s Ataxia”. No clinical trial involving epilepsy and RTA 408 was found in ClinicalTrials.gov.

AEBSF is a water-soluble, broad-spectrum, and irreversible inhibitor of serine proteases and can also avoid the activation of NADPH oxidase, the ROS generator [175,176]. In the perforant path stimulation model of status epilepticus, it was demonstrated that inhibition of excessive generation of ROS by AEBSF, significantly lessened seizure-induced cell death. These results indicate that NADPH oxidase inhibition can utilize as a new strategy of treatment to prevent brain damage in status epilepticus and related seizure disorders [50]. In a recent study, it was shown that combining AEBSF and RTA 408 in kainic acid-induced status epilepticus can reduce the occurrence of spontaneous seizures in animals following status epilepticus and modified the severity of epilepsy. It was also revealed that AEBSF and RTA 408 can reduce the generation of ROS through NADPH oxidase inhibition and increase the endogenous antioxidant system through Nrf2 activation respectively and preclude unwarranted ROS accumulation, mitochondrial membrane depolarization, and decrease neuronal death in *in vitro* seizure models [57]. No clinical trial was found regarding “brain disease and AEBSF” from ClinicalTrials.gov.

N-[3-(aminomethyl)benzyl] acetamidine or 1400 W, is an aralkylamine and acts as a highly selective inhibitor of iNOS [177]. With the kainic acid-induced status epilepticus, 1400 W, by decreasing the epileptiform spike rate in the first three days of after status epilepticus and can potentially modify the development of epileptogenesis and reduce the severity of chronic epilepsy [134]. Thus, 1400W is also revealed to modulate proteins involving neuroinflammatory responses such as heat shock protein beta-1, glial fibrillary acidic protein, and CD44 antigen in kainic acid-induced status epilepticus [178]. No clinical trial was found regarding “brain disease and 1400 W” from ClinicalTrials.gov.

Apocynin, a cell-permeable phenol, is recognized as a powerful inhibitor of NADPH oxidase [49]. It can block superoxide and peroxynitrite formation in murine macrophages [179] and increase glutathione synthesis via the activation of activation protein 1 (AP-1) [180]. In a pilocarpine-induced status epilepticus, it was demonstrated that apocynin reduced the production of ROS, lessened the extent of lipid peroxidation, diminished seizure-induced BBB disintegration, declined microglial activation and neutrophil infiltration, and further reduced neuronal death in the hippocampus [49]. This may suggest the therapeutic potential for apocynin in seizure-induced neuronal damage. No clinical trial was found regarding “brain disease and apocynin” from ClinicalTrials.gov.

AEOL10150, a metalloporphyrin with broad-spectrum catalytic antioxidant, possesses the ability to scavenge free radicals and inhibit lipid peroxidation [181,182,183]. It can cross the BBB to attain therapeutic concentrations in the brain, which is useful for this antioxidant to provide antioxidative capability sufficient to attenuate oxidative stress in pilocarpine-induced neurological damage and reduce mortality of experimental animals [135,136]. The beneficial effects depend on the ability of metalloporphyrin to scavenge peroxynitrite, lessen lipid peroxidation, with SOD and catalase-like activities [135]. Until now, no clinical trial was found about “brain disease and AEOL10150” from ClinicalTrials.gov.

Although this article focuses on the potential drugs and chemical compounds with the antioxidant character for status epilepticus, we need to stress that the treatment of choice for epilepsy is still the anti-epileptic drugs. However, taking into accounts the capability of antioxidant character in some anti-epileptic drugs, the choice of medications for epilepsy would add additional consideration and need to balance the gain and loss [151]. For example, different classes of antiepileptic drugs can influence the intima thickness of the common carotid artery in epileptic patients [184,185]. Several studies have shown that certain antiepileptic drugs exhibit antioxidant effects through modifying the activity of various enzymes system under epilepsy. A previous study showed that levetiracetam, a widely used anti-epileptic drug, exerted beneficial effects to reduce oxidative stress and lessen the severity of mitochondrial dysfunction after status epilepticus [83]. Phenytoin was found to have a neuroprotective effect over survival-promoting and antioxidant genes such as Akt1 and glutathione reductase in a DNA microarrays study [186]. Topiramate can inhibit free radical production, attenuate lipid peroxidation, and supporting the antioxidant redox system to protect pentylenetetrazol-induced brain injury [187]. In a recent study, it was shown that children with valproic acid monotherapy has significant antioxidant effects and decreases seizure activity [188]. Valproic acid may increase levels of glutathione and exert its neuroprotective effect [189]. Zonisamide is useful in animal and human studies of epilepsy. Zonisamide exerts antioxidant character by scavenging hydroxyl and nitric oxide radicals [190]. These examples just remind us to think about the drug choice in a more broaden way if possible with extra benefits in patients with epilepsy.

## 5. Conclusions and Future Perspective

Epilepsy is a common neurological disease with a prevalence of 0.5–1.5% in the general population. Although antiepileptic drug or anticonvulsants is a standard treatment for patients with epilepsy, more than one-third of patients remain in poor control of seizure frequency, the so-called drug-resistant epilepsy even with the several new antiepileptic drugs used in recent decades. Status epilepticus, in patients with prolonged or continuous epileptic seizures, is often associated with significant morbidity and mortality. Emerging evidence reveals the significant role of ROS/RNS in status epilepticus. It is reasonable to speculate that besides antiepileptic drugs, the addition of antioxidants as a combination therapy may exert an additional beneficial effect, lessen the neuronal damage, and improve clinical outcomes. Here, we provide some evidence for the potential application of antioxidants in epileptic patients. However, more studies are still mandatory both pre-clinically and clinically in order to achieve this goal. Although mounting evidence revealed the success of antioxidants treatment in status epilepticus in pre-clinical studies as summarized in Table 1, the difficulty of translation into clinical practice is obvious from the information of CliniclTrials.gov. There are some molecules, such as ascorbic acid, α-tocopherol, coenzyme Q10, lipoic acid, and melatonin that may represent an endogenous defensive mechanism to counteract oxidative stress, and other chemical compounds or drugs need to deal with the capability to enter the BBB if used in diseases of the central nervous system including epilepsy. Other possible reasons for lack of efficacy in clinical trials may stem from the underlying pathophysiological mechanisms. Status epilepticus or refractory epilepsy may trigger multiple pathophysiological and biochemical changes, as shown in Figure 1. These cascades reveal that excessive oxidative stress and inflammatory response from status epilepticus can cause highly reactive ROS/RNS, impair mitochondrial function, reduce the ATP supply, result in the apoptotic process, and to neuronal death. The combination therapy with one more drug to aim at various targets such as together with antioxidant, anti-inflammatory drugs or medications altering apoptotic pathways may yield additional or even synergistic effects. However, the current drug trial mainly uses one drug only to compare with placebo-control. This may also explain the lack of efficiency in various clinical trials due to the failure to cope with the complexity of pathophysiological changes in various diseases including epilepsy. This problem is warranted to further explored in clinical trials in the future.

## Figures and Tables

**Table 1:** Selected references of drugs or chemical compounds with antioxidant characters used in various animal model of status epilepticus. Information concerning clinical trials and various brain diseases including epilepsy and these drugs/chemical compounds from ClinicalTrials.gov are provided. (Abbreviations: Traumatic brain injury, TBI; Alzheimer disease, AD; Parkinson disease, PD; Huntington’s disease HD; Peroxisome proliferator-activated receptor gamma, PPARγ; Ataxia-telangiectasia, AT; Malondialdehyde, MDA; Superoxide dismutase, SOD; Glutathione peroxidase, GSH-Px; progressive supranuclear palsy, PSP; Thiobarbituric acid reacting substances, TBARS; Attention deficit hyperactivity disorder, ADHD; Inducible nitric oxide synthase, iNOS).

Name	Relevant Antioxidative Effects or Mechanisms	Pre-Clinical Models	Reference	Relevant Information about Brain Diseases from ClinicalTrials.Gov
N-acetylcysteine	mimetic of glutathione	pentylenetetrazol -induced model	[113]	Trials with neurodegenerative diseases, TBI, brain tumor, and vascular diseases. With one clinical trial concerning epilepsy (ClinicalTrials.gov Identifier: NCT02054949)
Ubiquinone (CoQ10)	potent antioxidant potentiates the antiepileptic effects of phenytoin treatment restores mitochondrial enzyme complex activities, reduce inflammation	pilocarpine-induced model pentylenetetrazol-induced model	[114,115]	Trials with migraine, stroke, AD, PD, HD, epilepsy, ataxia, mitochondrial disease. With one clinical trial concerning epilepsy (Identifier: NCT04488172)
Naringenin	recovery of glutathione content and antioxidant enzymes activity	pilocarpine-induced model	[116]	No clinical trial was found.
EUK-134	synthetic of superoxide dismutase/catalase mimetic, prevent excitotoxic neuronal injury	kainic acid-induced model	[117]	No clinical trial was found.
pioglitazone	PPAR γ agonist reduction of inflammatory responses increased UCP2 expression, reduced oxidant overproduction and improved mitochondrial function	genetically epileptic EL mice kainic acid-induced model	[118,119]	Trials with neurodegenerative diseases, stroke, brain tumor, atherosclerosis, adrenomyeloneuropathy, Friedreich’s Ataxia, and AT. No clinical trial involving epilepsy was found.
Aryl semicarbazides	reduced formation of MDA and increased formation of SOD and GSH-Px	pentylenetetrazol -induced model	[120]	No clinical trial was found.
α-lipoic acid	reduced lipid peroxidation level, nitrite content, augmented the SOD, catalase and GSH-Px activities	pilocarpine-induced model	[121,122]	Trials with AD, PD, mucopolysaccharidosis disorders, ischemic stroke, brain injury, adrenomyeloneuropathy, and PSP. No clinical trial concerning epilepsy was found.
Melatonin	scavenger of hydroxyl radical decreased nitrite content	kainic acid-induced model pentylenetetrazole -induced model	[123,124]	Trials with neurodegenerative diseases, TBI, brain tumor, stroke, birth-related brain injury, migraine, sleep disorders. Four trials concerning “epilepsy and melatonin” were found (Identifier: NCT02195661, NCT00965575, NCT01161108, and NCT01370486)
α-tocopherol (vitamin E)	oxidative stress with chaperone-mediated autophagy decreased TBARS production and total protein carbonylation	pilocarpine-induced model pentylenetetrazol- and methylmalonic acid-induced model	[125,126]	Trials with premature birth-related intraventricular hemorrhage, migraine and sleep disorders. No clinical trial concerning epilepsy was found.
Ascorbic acid (Vitamin C)	decreased lipid peroxidation and increased catalase activity increase GSH level and the decrease in lipid peroxidation level	pilocarpine-induced model penicillin -induced model	[127,128]	Trials with AD, PD, stroke, brain tumor, carotid atherosclerosis, ischemic or, hepatic encephalopathy, cerebral palsy, and ADHD. One study concerning epilepsy (Identifier: NCT02369822)
Selenium and sildenafil	lipid peroxides and nitrotyrosine levels, concomitantly with iNOS inhibition, normalization of TrxR activity and HO-1 expression, and evident neo-angiogenesis	pentylenetetrazol -induced model	[129]	For selenium, trials with phenylketonurias, AD, HD, stroke, cerebral palsy. Two trials concerning epilepsy (Identifier: NCT01764516, NCT01795170)
Selenium and Topiramate	inhibiting free radical supporting antioxidant redox system	pentylenetetrazol -induced model	[130]	As above information
Resveratrol	reduced markers both of oxidative stress and mitochondrial dysfunction suppressing oxidative stress and inflammation	lithium-pilocarpine-induced model kainic acid-induced model	[131,132]	Trials with AD, PD, HD, Friedreich ataxia and hypoxic brain. No clinical trial concerning epilepsy was found.
RTA 408	KEAP1 inhibition, Nrf2 activation	kainic acid-induced model	[133]	One clinical trial concerning “brain disease and RTA 408” for Friedreich’s Ataxia”. No clinical trial involving epilepsy was found.
AEBSF	NADPH oxidase inhibitor	perforant path stimulation model	[50]	No clinical trial was found.
RTA 408+ AEBSF	KEAP1 inhibition, Nrf2 activation + NADPH oxidase inhibitor	kainic acid induced model	[57]	No clinical trial was found
1400W	selective inhibitor of inducible nitric oxide synthase	kainic acid-induced model	[134]	No clinical trial was found.
Apocynin	an NADPH oxidase assembly inhibitor	pilocarpine-induced model	[49]	No clinical trial was found.
AEOL10150	metalloporphyrin catalytic antioxidant, scavenges peroxynitrite, inhibits lipid peroxidation with SOD and catalase-like activities	pilocarpine-induced model	[135,136]	No clinical trial was found.
